# Kissing duodenal ulcer in a young adult presenting as upper gastrointestinal bleeding in a resource-limited setting: A case report and literature review

**DOI:** 10.1016/j.ijscr.2025.110942

**Published:** 2025-01-24

**Authors:** Chinonso Paul Shu, Chukwuemeka G. Nwegbu, Djomo Y.A. Ginette, James Allen Brown

**Affiliations:** Pan-African Academy of Christian Surgeons, Mbingo Baptist Hospital, Cameroon

**Keywords:** NSAIDs and steroids abuse, Upper gastrointestinal bleeding, Kissing duodenal ulcers, Case report

## Abstract

**Introduction:**

Upper gastrointestinal bleeding from kissing duodenal ulcers has been reported frequently in elderly patients taking non-steroidal anti-inflammatory drugs (NSAIDs) and steroids but rarely reported in young adults. We reviewed the literature and discussed the management.

**Presentation of case:**

We report a case of a 29-year-old Cameroonian male on over-the-counter NSAIDs and steroids, who presented with fatigue, acute abdominal pain, melena, and hematemesis. After resuscitation, esophagogastroduodenoscopy (EGD) revealed a Forest IIB duodenal bulb ulcer. Bleeding could not be controlled locally, necessitating laparotomy, at which time, anterior and posterior perforated duodenal ulcers sealed against the liver and pancreas were found, with active bleeding of the posterior ulcer. The bleeding vessel was over-sewn, followed by an antrectomy and Billroth II gastrojejunostomy. The patient was discharged on the eighth postoperative day and was followed up two weeks later with a satisfactory outcome.

**Discussion:**

Kissing duodenal ulcers may present in young adults with a history of NSAIDS and steroid use. Perforation may not always be apparent especially if sealed. Urgent EGD does not decrease mortality.

**Conclusion:**

As demonstrated in this index case report, early diagnosis and surgical intervention could be lifesaving in a resource-limited setting.

## Highlights

• Kissing duodenal ulcers are rare but possible even in young adults.
• Individuals on over-the-counter steroids and NSAIDs are at higher risk.
• Clinical signs of perforation must not always be present.
• Where urgent EGD cannot be done, can be done within 24 h.
• Early surgical management in resource-limited settings is life -saving.

## 1 Introduction

The advent of medications that effectively reduce gastric acid and eradicate *Helicobacter Pylori* (*H. pylori*) infections has significantly reduced complications requiring surgery for peptic ulcer disease (PUD) [1]. Nevertheless, PUD affects close to four million people annually and has a lifetime risk of 5–10 % in the general population [1,2]. Peptic ulcer is the erosion of the mucosal layer of the stomach and/or duodenum extending into muscularis. It is usually a failure of the defense mechanisms of the stomach or insults from external agents, the most common being the bacterium *H. pylori* [3]. Chronic use of NSAIDs and steroids is associated with an increased risk of PUD [4]. A condition of two ulcers on opposite sides of the duodenum or stomach is called a kissing ulcer, which is more common in the duodenum [5]. Bleeding is the most common complication of PUD [6]. The complications of PUD result in significant morbidity and mortality with mortality of perforation reported as high as 5–25 % [7]. Effective management in resource limited settings often presents significant challenges as access to all necessary interventions is usually constrained. We equally reviewed the literature and discussed the management to highlight the relevance of such cases to clinicians worldwide. The work has been reported in accordance with the SCARE criteria [8].

## 2 Case presentation

A 29-year-old patient presented with a one-week history of extreme fatigue and three days of melena and hematemesis. They complained of progressive, non-radiating epigastric pain, and anorexia. The patient had no fever or change in bowel habits. They were taking over-the-counter NSAID (Diclofenac) and steroids (Dexamethasone) for right lower extremity ulcer.

On examination, the patient looked acutely ill. Their vital signs were, blood pressure of 80/58 mmHg (mean arterial pressure of 65 mmHg); pulse of 124 beats/min; respiratory rate of 28breaths/min, oxygen saturation of 99 % on room air; temperature of 36.5 °C; Glasgow Coma Score of 15/15.

They were pale without jaundice. Their mucous membranes were dry.

Their Inferior vena cava measured 0.76 cm and completely collapsed during inspiration.

The patient's abdomen was flat and soft with mild epigastric tenderness and normal bowel sounds but no rebound tenderness or guarding. They had dark tarry stools on rectal examination.

They had a right medial malleolar ulcer of 6 cm in diameter, well-circumscribed with a necrotic floor. Chest exam revealed regular tachycardia, otherwise unremarkable. All peripheral pulses were palpable, though weak and thready.

Laboratory investigation revealed a critical hemoglobin level of 2.9 g/dl, a leukocytosis with neutrophilic predominance, and severe microcytic, hypochromic anemia. Stool *H. pylori* antigen test was negative. There was evidence of acute kidney injury (Table 1*).*

**Table 1:** Laboratory investigations on admission.

WBC(x10^9^/L)	HB(g/dl)	MCV(fl)	MCH(pg)	PLT(/μl)	Na(mEq/L)	K(mEq/L)	Cl(mEq/L)	Urea (mg/dl)	Creat (mg/dl)	Hpylori
24.69	2.9	70.6	22	503,000	140	4.45	105.8	52.09	1.73	Neg

Their diagnoses were hemorrhagic shock secondary to NSAID/steroid-associated upper gastrointestinal bleeding and a chronic right lower leg ulcer.

The patient was admitted to the intensive care unit, where they received an intravenous (IV) proton pump inhibitor (PPI), Omeprazole 80 mg stat, then 40 mg twice daily, and were resuscitated with IV fluids and two units of whole blood transfusions.

EGD the following day revealed fresh blood with clots in the stomach and a wide, deep duodenal bulb ulcer with a fresh clot in the duodenum (Forrest IIB). The second portion of the duodenum was not reached for fear of dislodging the clot.

**Fig. 1:**
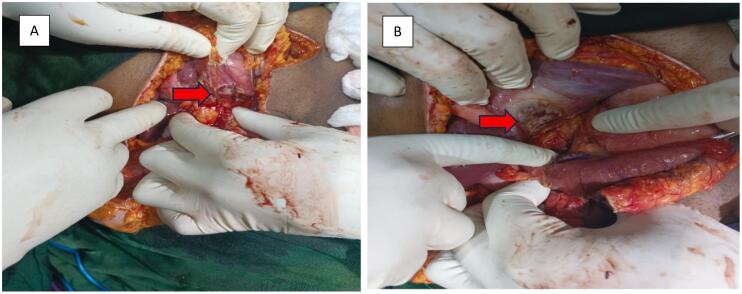
Intraoperative findings: A- the ligated vessel in the posterior wall, B- reveals anterior perforation with evidence of liver concealing.

The patient was still hemodynamically unstable. They received a gram of tranexamic acid before transfer to the operating room, where their abdomen was opened through an extended oblique right subcostal incision. The patient had an anterior 4-cm, perforated, anterior duodenal bulb ulcer sealed by the liver, and a 4-cm perforated posterior bulb ulcer with a bleeding vessel (Fig. 1*)*. There was minimal peritoneal contamination. Ligation of the bleeding vessel was followed with an antrectomy and Billroth II gastrojejunostomy. Less than 2-cm of proximal duodenum was mobilized with much difficulty because of peripancreatic inflammation and bleeding. The duodenal stump was closed in two layers and covered with an omental patch. The patient had an uneventful postoperative course.

During the patient's review visit two weeks later, they were doing well with no complaints. The surgical site had healed and the patient reported no hematemesis or melena. Counseling on the use of NSAIDs and steroids was reinforced.

## 3 Discussion

Kissing duodenal ulcers are rare, and, approximately one in ten patients who have surgery for bleeding, have an associated unsuspected perforation [9]. Also, patients with a combination of bleeding and perforation have a higher operative mortality compared to those with either complication [9]. Our patient had simultaneous sealed anterior and posterior duodenal bulb perforations, with a bleeding vessel in the posterior ulcer. The liver sealed the anterior perforation while the pancreas sealed the posterior. This explains why they did not have the classic triad of sudden onset abdominal pain, tachycardia and abdominal rigidity. Our literature search found no case report with this same configuration of pathology. The closest was a report by *Triantafyllou et al* of double perforated kissing ulcers of duodenum (not sealed) in a 46-year-old male, who presented with the classical triad [10]. Another report by *Vahedian et al* in Iran reported a sealed anterior perforated duodenal ulcer and a hemorrhagic posterior ulcer in a 27-year-old male for which they were able to ligate the bleeder and did a pyloroplasty and vagotomy [11]. There is limited published data on the incidence of peptic ulcer perforations, especially in young patients in Sub-Saharan Africa [12]. The use of corticosteroids is associated with gastrointestinal bleeding and perforation, and concurrent use of both NSAIDs and steroids is related to gastroduodenal perforation [4,13]. Perforation is more common in self-prescribed use or abuse of NSAIDs [14] especially when not on PPI which was the case in our patient.

The recommended treatment for perforated duodenal ulcers varies depending on the size, anatomical location, involved organ, hemodynamic stability of the patient and available resources including the surgeon's skill [[10], [11], [12], [13], [14]]. Simple closure and omentopexy with no need for acid-reducing procedures are recommended for simple perforations in unstable patients in this era of proton pump inhibitors [12]. Management of bleeding duodenal ulcer depends on what is available technically, ranging from, endoscopic injection, clipping, CT angiogram with embolization, open surgical ligation of bleeding vessel or resection and drainage procedures [15]. Endoscopic management of bleeding is usually successful in about 75 %, with rebleeding still managed endoscopically in most cases [11]. However, a randomized controlled trial by *Lau et al*, revealed urgent endoscopy (within six hours of consultation) did not decrease mortality in patients with high risk of rebleeding and death [16]. So, it is reasonable to resuscitate patients and get endoscopy within 6–24 h if it cannot be done immediately. This approach is relevant in our setting, where endoscopy is not available 24/7. Our facility does not have an intervention radiologist for embolization. Although meta-analyses are conflicting with regards to the use of tranexamic acid in UGIB, guidelines do not recommend it's use, unless in clinical trials [17]. In settings like ours with limited management options, adaptation of guidelines is necessary, especially when an option is not contraindicated. This explains why our patient received a stat dose of tranexamic acid.

Our patient presented in hemorrhagic shock with double duodenal sealed perforation and a bleeding posterior ulcer and almost no normal first part of the duodenum. Their pathology necessitated resection of the proximal duodenum and gastric antrum with a Billroth II reconstruction. Other surgical options include wedge resection, antrectomy, Billroth I gastroduodenostomy, Roux-en-Y reconstruction, and pyloroplasty with or without vagotomy [18]. Truncal vagotomy has become less common due to its complexity and high morbidity, especially with the availability of PPIs [18].

A rare but serious complication of this procedure is a duodenal stump leak, which has a mortality rate of up to 12 %. Techniques to prevent this complication include avoiding excessive duodenal skeletonization, careful stitch placement to prevent ischemia, purse-string closure, omental patching, and even jejunoduodenostomy [19,20]. Management options for a duodenal stump leak include resuscitation, percutaneous drainage, abdominal exploration, and possible washout. If necessary, further closure attempts can be made after decompression, ensuring any distal obstructions are relieved [20].

## 4 Conclusion

Though not a frequent finding, clinicians should maintain a high index of suspicion for perforation in patients on NSAIDs and steroids who present with upper gastrointestinal bleeding even in the absence of clinical evidence of perforation. Early surgical intervention could be indicated in a resource-limited setting if nonsurgical interventions are unavailable.

## Abbreviations

EGD: Esophagogastroduodenoscopy
NSAIDs: Non-steroidal Anti-inflammatory Drugs
MCV: Mean Corpuscular Volume
PLT: Platelets
H. pylori: Helicobacter Pylori
WBC: White Blood Count
HB: Hemoglobin
UGIB: Upper gastrointestinal bleeding
PPI: Proton Pump Inhibitors

## CRediT authorship contribution statement

CPS and CGN managed the patient and conceived the idea. CPS and DYAG collected the data. CPS, CGN and DYAG wrote the initial manuscript. CPS, CGN and JAB read and corrected the whole manuscript. All authors approve of this manuscript submitted.

## Ethical approval

Ethical clearance was gotten from the Cameroon Baptist Convention Health Board Institutional Review Board with ref.: IRB2023-84.

## Guarantor

Chinonso P. Shu

## Consent for publication

Written informed consent was obtained from the patient for publication of this case report and any accompanying images. A copy of the written consent is available for review by the Editor-in-Chief of this journal.

## Funding

This research did not receive any specific grant from funding agencies in the public, commercial or not-for-profit sectors.

## Registration of research studies

Not applicable.

## Declaration of competing interest

None.

## Data Availability

This is a case study so all data used are within the article.
